# Optimization of scan protocol for high temporal resolution magnetic resonance imaging of the liver under single breath-holding using compressed sensing and parallel imaging techniques in a 1.5-T magnetic resonance system

**DOI:** 10.1259/bjro.20210018

**Published:** 2021-09-29

**Authors:** Fumiaki Fukamatsu, Akira Yamada, Hayato Hayashihara, Yoshihiro Kitou, Yasunari Fujinaga

**Affiliations:** ^1^ Department of Radiology, Shinshu University School of Medicine, Matsumoto, Japan; ^2^ Division of Radiology, Shinshu University Hospital, Matsumoto, Japan

## Abstract

**Objective:**

To optimize the scan protocol for high temporal resolution magnetic resonance (MR) imaging of the liver under single breath-holding, using compressed sensing (CS) and parallel imaging (PI) techniques in a 1.5 T MR system.

**Methods:**

31 healthy volunteers who underwent fat-suppressed gradient-echo *T*_1_ weighted imaging using a 1.5 T MR system were included. Image quality was evaluated on altering various imaging parameters in CS and PI so that the scan time was adjusted to 10 and 6 s within a single breath-holding. Normalized standard deviation (nSD = SD/mean value) and signal-to-noise ratio (SNR = mean value/SD) of liver signal intensity were measured. Visual scores for the outline of the liver and inferior right hepatic vein (IRHV) were evaluated using a 4-point scale and compared with that of the reference standard (20 s scan without CS).

**Results:**

The nSD and SNR were not significantly different when the 10 s scan with CS factor 2.0 and the 6 s scan with CS factor 2.0 and 2.5 were compared to the 20 s scan. Overall visual score (mean score of the outline of the liver and IRHV) was significantly better (*p* < 0.05) with the 10 s scan with CS factor 2.0 compared to the other scan protocols.

**Conclusion:**

The 10 s scan with CS factor 2.0 should be recommended for high temporal resolution MR imaging of the liver using CS and PI in a 1.5 T MR system.

**Advances in knowledge:**

This study conducts a novel MR imaging of the liver using CS and PI in a 1.5 T MR system.

## Introduction

Multiple arterial phase imaging is useful for the detection of hepatocellular carcinomas.^1–4^ High temporal resolution magnetic resonance (MR) imaging is important for obtaining optimal arterial phase images in hepatic dynamic contrast-enhanced MR imaging (DCE-MRI) under single breath-holding. Moreover, an association has been described between the intravenous bolus injection of gadoxetate disodium and transient severe motion in the arterial phase, and it has been reported that the use of multiple arterial phase imaging minimizes the effect of transient severe motion during gadoxetate disodium-enhanced liver MR imaging.^5,6^ Accelerated data acquisition techniques are necessary to obtain multiple arterial phases in MR systems.

Many studies have evaluated the utility of several techniques such as time-resolved imaging, compressed sensing (CS), and parallel imaging (PI) in hepatic DCE-MRI to accelerate data acquisition. Complementary use of these techniques was useful for reducing the acquisition time with a 3 T MR system.^7–9^ In fact, some authors have succeeded in obtaining MR images of the liver under single breath-holding with the complementary use of CS and PI in a 3 T MR system.^8,9^ Better image quality can be obtained by using image reconstruction of both CS and PI together than using image reconstruction of PI alone.^10,11^ When the acquisition time is the same in both MR systems, the signal-to-noise ratio (SNR) and spatial resolution are lower for a 1.5 T MR system than for a 3 T MR system. On the other hand, motion artifacts and metal artifacts are less with a 1.5 T MR system. In a 3 T MR system, since the radiofrequency (RF) penetration declines and becomes non-uniform, the image quality degrades in the case of a large volume of ascites.^12^ Therefore, we considered liver MR imaging with a 1.5 T MR system to be useful.

The 1.5 T MR system is widely used; however, no study using a 1.5 T MR system with a combination of CS and PI has been conducted to date. Thus, we aimed to optimize the scan protocol for high temporal resolution MR imaging of the liver under single breath-holding with a 1.5 T MR system using a combination of CS and PI.

## Methods and materials

### Participants

This study was approved by our institutional review board. Informed consent was obtained from all the participants. 31 consecutive healthy volunteers (19 men and 12 women), with a mean age of 36 years, were enrolled in our study. We recruited volunteers who met the following criteria at Shinshu University Hospital (Matsumoto, Japan) from October to December 2017. The inclusion criteria were being healthy and without underlying illness. The exclusion criteria were as follows: (1) abnormal findings on MRI, such as liver tumors and ascites, (2) image quality degradation due to poor breath-holding and body movement, and (3) contraindications to MRI, such as metallic implants. All volunteers satisfied the inclusion criteria, and none met the exclusion criteria.

### MR imaging

MR imaging was performed using a 1.5 T scanner (Optima MR450w; GE Healthcare, Waukesha, WI) equipped with a 30-channel cardiac and spine coil. All participants underwent axial fat-suppressed gradient-echo *T*_1_ weighted imaging—liver acquisition with volume acceleration (LAVA) using a combination of CS (CS additional acceleration) and PI (Auto-calibrating Reconstruction for Cartesian imaging; ARC, data-driving parallel imaging reconstruction). LAVA is a three-dimensional spoiled gradient-echo sequence used for dynamic contrast-enhanced abdominal imaging with high SNR. The trajectory of data sampling in k-space was Cartesian. Imaging parameters were as follows: field of view = 320×320 mm^2^, slice thickness = 4 mm, matrix = 256×192, bandwidth = 125 kHz, repetition time = 5.546 ms, echo time = 1.416 ms, and flip angle = 12°.

Three scan times of LAVA were used: 20 s, 10 s, and 6 s. For the reference standard, a 20 s scan without CS was used. The 10 s and 6 s scans were used to obtain double or triple arterial phase DCE-MRI in a clinical case study. The scan time of LAVA was adjusted within a single breath-holding by changing the combination of CS and PI factors (Table 1). The product of phase ARC and slice ARC was defined as the total PI factor. The 31 volunteers were divided into two groups: a 6 s scan group and a 10 s scan group. A total of 18 (10 men, 8 females) and 13 (9 men, 4 women) participants were included in the 6 s and 10 s scan groups, respectively. Both groups underwent a 20 s scan.

**Table 1.** The scan time and acceleration factor for each scan protocol

Scan time	CS factor		PI factor	
		Phase ARC	Slice ARC	Total PI factor^*a*^
20 s	1.0	2.0	1.0	2.0
10 s	1.2	2.0	2.0	4.0
	1.55	2.0	1.55	3.1
	2.0	2.0	1.2	2.4
6 s	1.5	2.0	3.0	6.0
	2.0	2.0	2.0	4.0
	2.5	1.5	2.0	3.0

ARC, Auto-calibrating reconstruction for cartesian imaging; CS, Compressed sensing; PI, Parallel imaging.

a The product of phase ARC and slice ARC.

### Image analysis

Two board-certified radiologists who had 16 and 6 years of experience in abdominal imaging drew the regions of interest (ROIs) of the liver according to the following criteria: location, the posterior segment of the right hepatic lobe; level, the posterior segmental branch of the portal vein; and large vessels were not included. The mean value and standard deviation (SD) within the ROIs were measured and the normalized SD (nSD = SD/mean value) and SNR (mean value/SD) of the liver were obtained. SNR was obtained by the method using the same image.^13,14^

**Figure 1.**
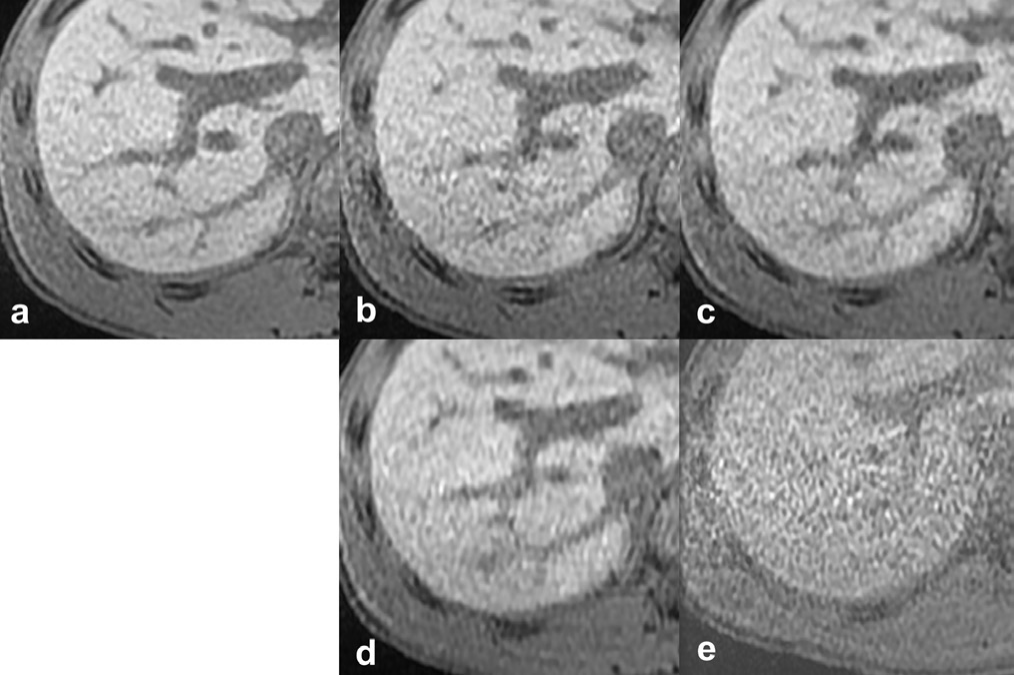
Reference images of the outline of the liver. The *vs*), scan time, CS factor, and total PI factor of each reference image are: (**a**) reference standard, 20 s scan, (**b**) *vs* 4, 10 s scan, CS 1.2, PI 4.0, (**c**) *vs* 3, 10 s scan, CS 1.55, PI 3.1, (**d**) *vs* 2, 6 s scan, CS 2.0, PI 4.0, (**e**) *vs* 1, 6 s scan, CS 1.5, PI 6.0. CS, compressed sensing; PI, parallel imaging; vs, visual score.

**Figure 2.**
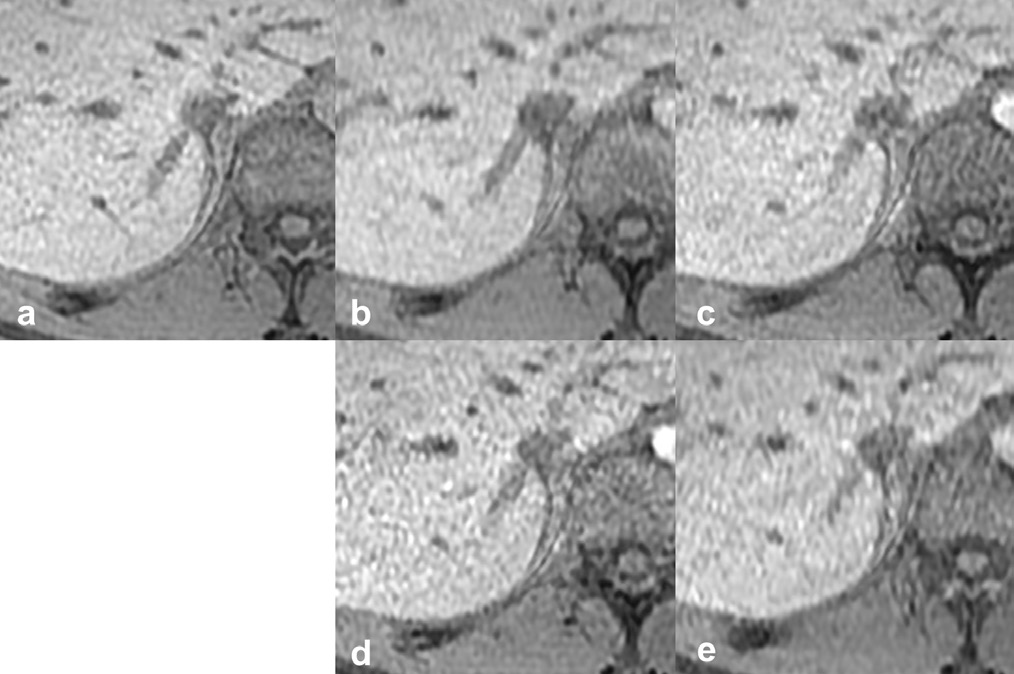
Reference images of the outline of the IRHV. The *vs*, scan time, CS factor, and total PI factor of each reference image are: (**a**) reference standard, 20 s scan, (**b**) *vs* 4, 10 s scan, CS 2.0, PI 2.4, (**c**) *vs* 3, 10 s scan, CS 1.55, PI 3.1, (**d**) *vs* 2, 10 s scan, CS 1.2, PI 4.0, (**e**) *vs* 1, 6 s scan, CS 2.0, PI 4.0. CS, compressed sensing; IRHV, inferior right hepatic vein; PI, parallel imaging; vs, visual score.

**Figure 3.**
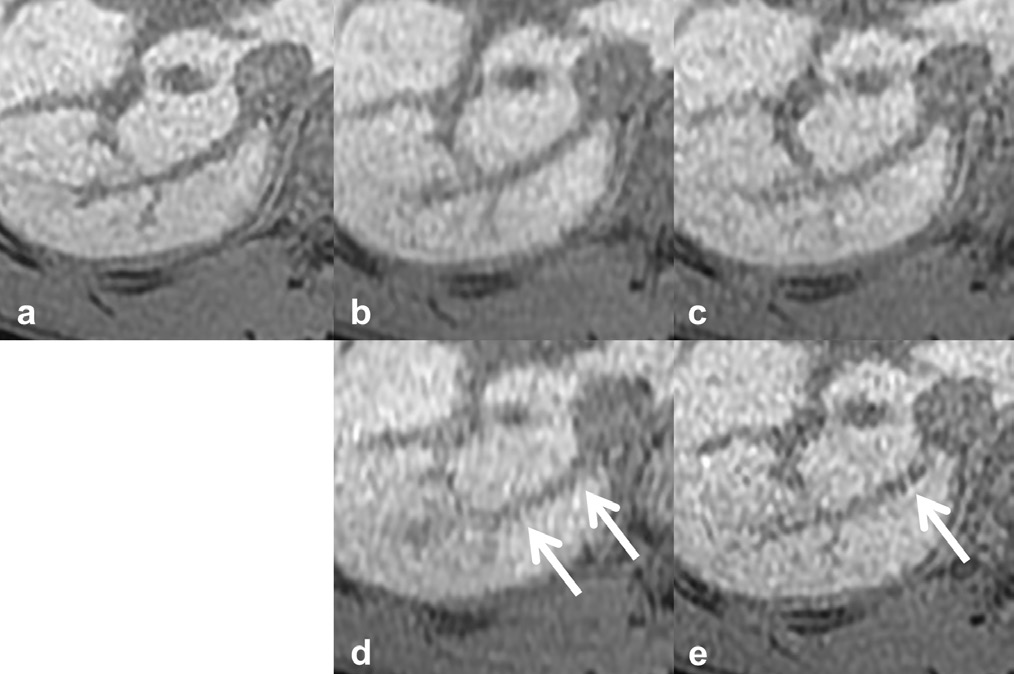
Reference images of continuity of the IRHV. The *vs*, scan time, CS factor, and total PI factor of each reference image are: (**a**) reference standard, 20 s scan, (**b**) *vs* 4, 10 s scan, CS 2.0, PI 2.4, (**c**) *vs* 3, 10 s scan, CS 1.55, PI 3.1, (**d**) *vs* 2, 6 s scan, CS 2.0, PI 4.0, (**e**) *vs* 1, 10 s scan, CS 1.2, PI 4.0. The continuity of IRHV vanished (arrows). CS, compressed sensing; IRHV, inferior right hepatic vein; PI, parallel imaging; vs, visual score.

**Figure 4.**
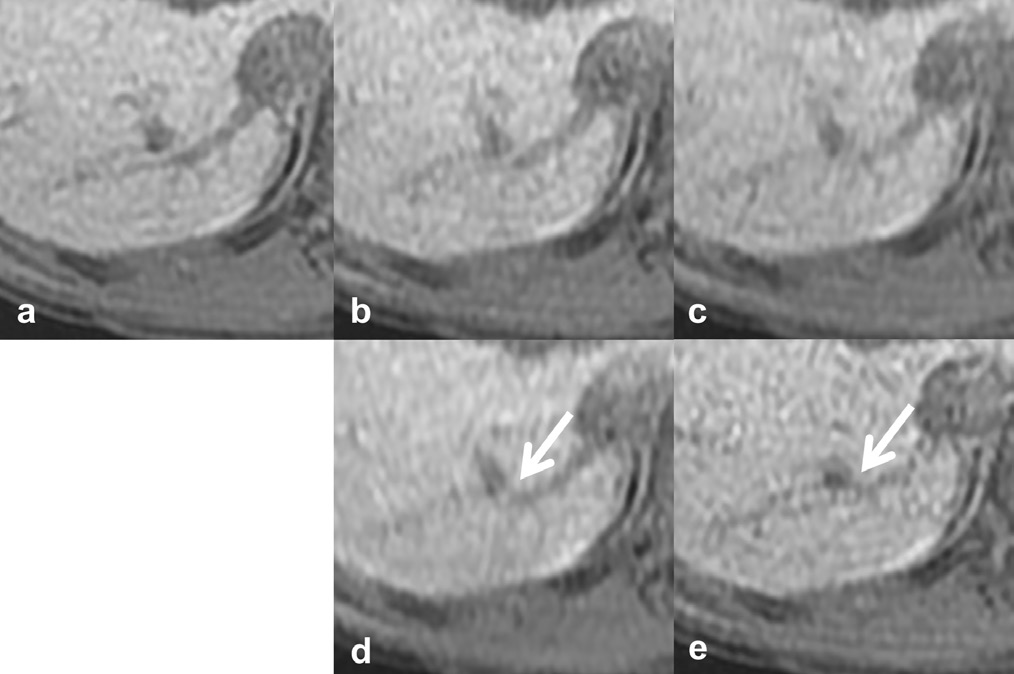
Reference images of pseudo structures of the IRHV. The *vs*, scan time, CS factor, and total PI factor of each reference image are: (**a**) reference standard, 20 s scan, (**b**) *vs* 4, 10 s scan, CS 1.55, PI 3.1, (**c**) *vs* 3, 10 s scan, CS 2.0, PI 2.4, (**d**) *vs* 2, 6 s scan, CS 2.0, PI 4.0, (**e**) *vs* 1, 10 s scan, CS 1.2, PI 4.0. There was continuity between the IRHV and other vessels, as if a portal venous shunt (arrows). CS, compressed sensing; IRHV, inferior right hepatic vein; PI, parallel imaging; vs, visual score.

The following four items were scored using the visual score ( *vs* ) (Table 2): (a) outline of the liver (location: the posterior segment of the right hepatic lobe, level: posterior segmental branch of the portal vein), (b) outline of the inferior right hepatic vein (IRHV), (c) continuity of the IRHV, and (d) pseudo-structures of the IRHV. Pseudo structures were defined as features not seen on 20 s scan, as if a portal venous shunt. A 4-point scale was used for *vs* , and we defined the *vs* as shown in Table 2 and Figures 1–4. The vanishing of the continuity of IRHV were defined as *vs* 1. The *vs* was evaluated by the two board-certified radiologists who were mentioned before.

**Table 2.** Visual scores of evaluation items for qualitative analysis of the images

Evaluation items		Visual score		
	4	3	2	1
A–C	equivalent to 20 s scan	relatively good	relatively poor	poor
D	no pseudo structures	probably no pseudo structures	probably pseudo structures exist	pseudo structures exist

A, Outline of the liver; B, outline of the inferior right hepatic vein (IRHV); C, continuity of the IRHV; D, pseudo-structures of the IRHV.

### Statistical analysis

For the quantitative evaluation, the mean nSD and SNR of each scan protocol in the two groups were compared to those of the 20 s scan using a *t*-test, and the correlation coefficient between image qualities (nSD and SNR) and acceleration factors (total PI factor and CS factor) was evaluated. For the qualitative evaluation, the null hypothesis, which states that the *vs* was smaller than 3 (relatively poor image quality compared to 20 s scan), was tested using a Mann–Whitney *U* test. The Overall *vs* (mean score of the sum of four items) was also evaluated. Intraclass correlation coefficient (ICC) was calculated for nSD, SNR, and *vs* statistical significance was defined as a *p* value < 0.05. All statistical analyses were performed using MATLAB 2018a (Mathworks, Natick, MA).

## Results

There was a significantly strong agreement in nSD (ICC = 0.867, *p* < 0.0001) and SNR (ICC = 0.831, *p* < 0.0001) measurements between the two radiologists. The mean nSD and SNR of the 20 s, 6 s, and 10 s scan, and the respective CS factor and total PI factor are shown in Table 3. The mean nSD of the liver was significantly higher compared to that of the 20 s scan (reference standard) when a 6 s scan with CS factor 1.5 (nSD = 0.188) and 10 s scans with CS factor 1.2 (nSD = 0.107) and 1.55 (nSD = 0.092) were used. No significant difference in the mean nSD compared to that of the 20 s scan was observed when a 6 s scan with CS factor 2.0 (nSD = 0.087) and CS factor 2.5 (nSD = 0.080), a 10 s scan with CS factor 2.0 (nSD = 0.077) were used. SNR exhibited a similar trend with nSD. The image noise significantly increased in correlation with increasing total PI factor to maintain the scan time.

**Table 3.** The mean nSD and SNR, and the respective acceleration factor of each scan protocol in two groups

	6 s scan group (×3)				10 s scan group (×2)			
Scan time	6 s			20 s	10 s			20 s
CS factor	1.5	2.0	2.5	1	1.2	1.55	2.0	1
nSD^*a*^	0.188^*c*^	0.087	0.080	0.088	0.107^*c*^	0.092^*c*^	0.077	0.077
SNR^*b*^	5.504^*c*^	11.69	12.84	11.79	9.668^*c*^	11.11^*c*^	13.37	13.39
Total PI factor	6.0	4.0	3.0	2.0	4.0	3.1	2.4	2.0

CS, compressed sensing; PI, parallel imaging; SNR, Signal-to-noise ratio; nSD, Normalized standard deviation.

a SD/mean value.

b Mean value/SD,

c The score was significantly different from that of the 20-s scan (*p* < 0.05).

**Figure 5.**
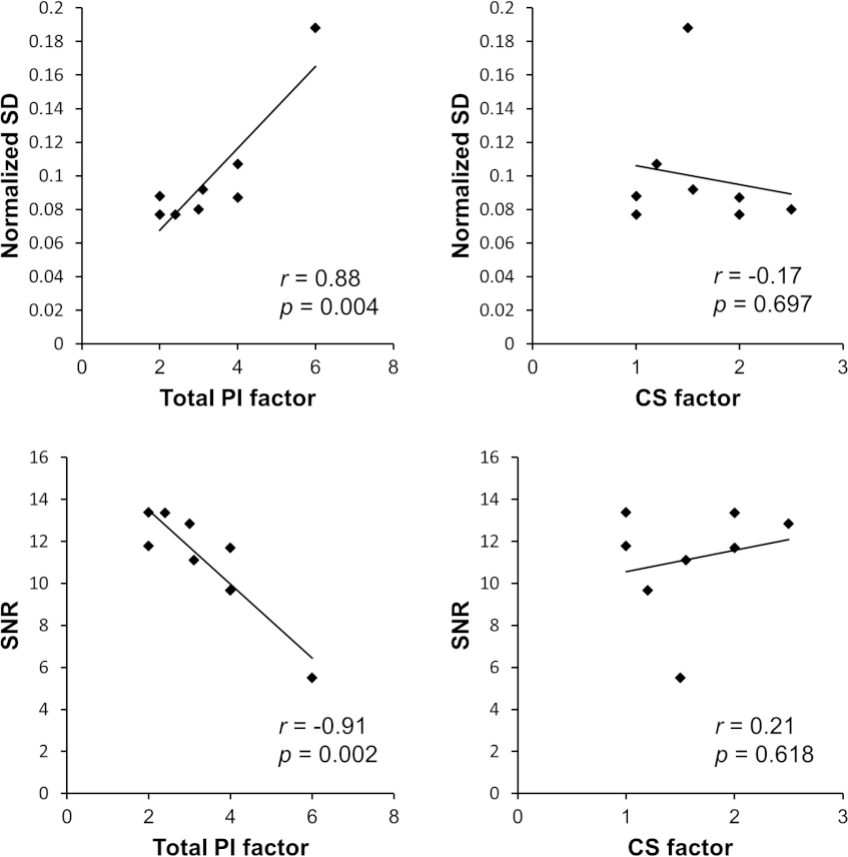
Correlations between image qualities (nSD and SNR) and acceleration factors [(total PI factor and CS factor). CS, compressed sensing; PI, parallel imaging; SD, normalized standard deviation; SNR, signal-to-noise ratio.

The correlations between the image qualities (nSD and SNR) and the acceleration factors (total PI factor and CS factor) are shown in Figure 5. The correlation coefficient of nSD was 0.88 (*p* = 0.004) for the total PI factor and −0.17 (*p* = 0.697) for the CS factor, and that of SNR was −0.91 (*p* = 0.002) for the total PI factor and 0.21 (*p* = 0.618) for the CS factor. A significant correlation was observed between the total PI factor and the image qualities.

There was a significantly strong agreement in *vs* evaluation between the two radiologists (ICC = 0.808, *p* < 0.0001). The *vs* of the four items in the 6 s and 10 s scans and the respective CS factor and total PI factor are shown in Table 4. When a 6 s scan was used, the *vs* of the outline and continuity of IRHV were significantly smaller than 3, regardless of the CS factor. The *vs* of the pseudo-structures with a 6 s scan was significantly smaller than 3, when the CS factor larger than 2.0 was used. The *vs* of the outline of the liver and IRHV with a 10 s scan was not significantly smaller than 3, regardless of the CS factor. The overall *vs* (mean score of the four items) was not significantly smaller than three only in the 10 s scan with CS factor 2.0. When the CS factor was 2.0 in the 10 s scan, the loss of image quality with a *vs* less than three was not seen in any of the items, and the total PI factor was the lowest (2.4) among the various protocols that were tested in this study.

**Table 4.** Visual score and the respective acceleration factor of each scan protocol

	CS factor (6 s scan)			CS factor (10 s scan)		
	**1.5**	**2.0**	**2.5**	**1.2**	**1.55**	**2.0**
A: Outline (Liver)	2.4	2.0^*b*^	1.9^*b*^	3.5	3.4	2.9
B: Outline (IRHV)	2.2^*b*^	1.7^*b*^	1.7^*b*^	3.0	2.5	2.8
C: Continuity (IRHV)	1.3^*b*^	1.3^*b*^	1.5^*b*^	2.3^*b*^	3.0	3.2
D: Pseudo-structures (IRHV)	3.2	1.9^*b*^	2.2^*b*^	2.5^*b*^	2.1^*b*^	3.1
Overall^*a*^	2.3^*^	1.7^*^	1.8^*^	2.5^*^	2.8^*^	3.0
Total PI factor	6.0	4.0	3.0	4.0	3.1	2.4

CS, Compressed sensing; IRHV, Inferior right hepatic vein; PI, Parallel imaging.

a Mean score of (A + B + C + D).

b The score was significantly smaller than 3 (*p* < 0.05)

## Discussion

In our study, the best image quality was observed in the 10 s scan with a CS factor of 2.0 performed using a 1.5 T MR system. The image noise was significantly increased in correlation with increasing total PI factor to maintain scan time. It is known that the image quality degrades as the PI factor increases.^11,15^ There was no significant correlation between the CS factor and image noise. We considered that the image quality in a 1.5 T MR system was mainly determined by the total PI factor (phase*slice ARC), even if the PI and CS were used together. This can be explained by the impaired signal reproduction in CS reconstruction owing to the increased noise by PI.

Several studies have shown the clinical usefulness of high temporal resolution abdominal MR imaging using a combination of CS and PI in a 3 T MR system. Zhang et al^16^ showed the feasibility of fast pediatric three-dimensional free-breathing DCE-MRI with high scan efficiency (scan time, 6.5 s) and image quality similar to respiratory-triggered acquisition. However, in this study, the image quality in the 6 s scan degraded significantly in a 1.5 T MR system compared to that in the 20 s scan, and clinically sufficient image quality was not observed. We consider that a scan time equal to that of a 3 T MR system leads to loss of image quality in a 1.5 T MR system since the SNR of a 1.5 T MR system is lower than that of a 3 T MR system. Therefore, it may be necessary to employ other acceleration techniques, such as view sharing, to maintain the image quality equivalent to a 3 T MR system, while the scan time remains the same.

Pseudo-structures, defined as features not seen on 20 s scan (reference standard), were observed in our study. Aliasing artifacts are known as artifacts related to PI. A specific artifact related to CS has not been reported, and it has been reported that CS reduces motion artifacts.^9^ In a previous study that evaluated the image quality of a combination of CS and PI in a 3 T MR system, the presence of in-plane aliasing artifacts, motion artifacts, and fat suppression deficiency were evaluated; however, pseudo-structures were not mentioned.^8^ Therefore, we considered pseudo-structures to be artifacts different from the ones previously reported, such as aliasing, motion, and susceptibility artifacts. In our study, the *vs* of the pseudo-structures in the 6 s scan with the CS factor larger than 2.0 was significantly smaller than 3, on the other hand, that of the 10 s scan with the CS factor smaller than 2.0 (relatively high total PI factor) was significantly smaller than 3. We suspected that the pseudo-structures were the originally non-existent structures and the errors produced by CS reconstruction due to the low SNR in a 1.5 T MR system or the increased noise by PI.

There were some limitations in our study. First, the number of participants was small, and participants of the 10 s scan group were different from that of the 6 s scan group. These might have affected the results. Second, the participants of this study were limited to healthy volunteers without liver diseases. Liver MR imaging is often targeted in patients with liver diseases such as liver failure, liver cirrhosis, and hepatocellular carcinomas; therefore, our results should be validated in such patients. However, our results provide the first evidence of a practical solution for performing high temporal resolution liver MR imaging using PI and CS in a 1.5 T MR system. We believe our data will promote future applications of high temporal resolution liver MR imaging for patients with liver diseases. Third, hepatic DCE-MRI was not performed. It is not clear if our proposed protocol is effective not only in non-contrast MR imaging but also in DCE-MRI because contrast media can produce strong tissue contrast even if a 1.5 T MR system is used. Future validations are needed to elucidate whether our proposed protocol is clinically useful for high temporal resolution liver MR imaging dedicated to specific clinical situations such as the detection of hepatocellular carcinomas.

## Conclusion

A 10 s scan with a CS factor of 2.0 should be recommended for high temporal resolution MR imaging of the liver using CS and PI in a 1.5 T MR system. The image quality in a 1.5 T MR system appears to be mainly determined by the total PI factor (phase*slice ARC), even if PI and CS are used together.
